# And the stars look down: science beyond the finite: From prehistorical science to postscience

**DOI:** 10.1038/s44319-024-00202-w

**Published:** 2024-07-05

**Authors:** Vladimir Leksa

**Affiliations:** Laboratory of Molecular Immunology, Institute of Molecular Biology, Slovak Academy of Sciences, Bratislava, Slovakia

**Keywords:** Economics, Law & Politics, Evolution & Ecology, Science Policy & Publishing

## Abstract

Modern science has brought undisputable welfare to mankind, but also harmful consequences, and it has to face great challenges to find a way out of the current global crisis.

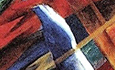

If you visit the sandy beaches of Namibia, you may notice people hounding seals. They spot their target with binoculars, then run across the sand and finally catch a seal with something that resembles a butterfly net. But they are not hunters. With knives, tongs or scissors, they skilfully remove plastic shackles from the seals—human waste that pollutes the oceans. As pups, seals become entangled into discarded fishing nets, plastic beer can holders and other things, and as they grow, the achievements of human civilisation cut deeper and deeper into their skin. The rescue team from the Ocean Conservation Namibia Organization (www.ocnamibia.org/) save dozens of seals every day (Curtis et al, 2021).

An expression of the human intellect is science, which has brought enormous welfare to humans; now, science must bring welfare to all living beings on Earth.

Animals severely injured by humans are a fitting image of the ecological damage that mankind has wrought on the environment (Fig. 1). The biologist Jane Goodall, who spent decades studying chimpanzees in the heart of Africa, has, for a long time, drawn attention to the violence that we commit against our closest relatives (Ross et al, 2008)—the common ancestor of humans and chimpanzees lived at the end of the Miocene, roughly 9 million to 6 million years ago (Almecija et al, 2021). Goodall is not losing hope, however. In her *Book of Hope*, she identifies human intellect as one of the reasons for her optimism: that, eventually, we might find a more sustainable and kind way to live. An expression of the human intellect is science, which has brought enormous welfare to humans; now, science must bring welfare to all living beings on Earth.

**Figure 1:**
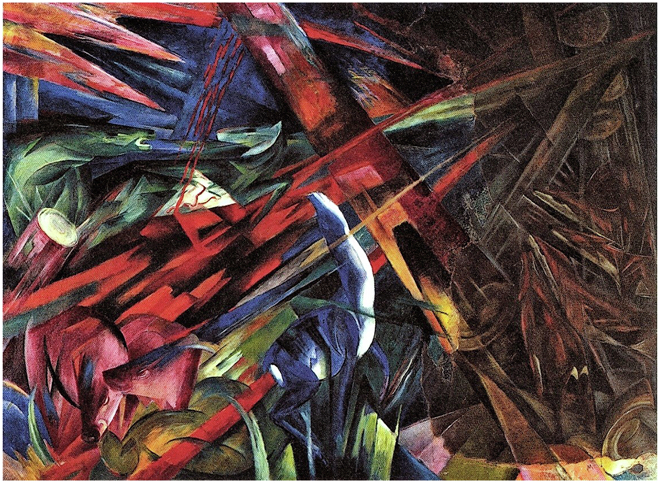
*Tierschicksale* (Fate of the animals) 1913 by Franz Marc. The right part of the painting was damaged in a warehouse fire in 1916 after Marc’s death and was later restored by Paul Klee using old photographs. Oil on Canvas. Wikimedia/Public Domain.

## The other side of the prism

One characteristic of our species is the ability to make artefacts. We do not know the names of prehistorical inventors who tamed fire, designed flint tools or invented the wheel and the plough, but we know that their work has changed the face of Earth. Through these changes, we had been passing through interfaces: our abilities and our options greatly expanded each time similar to white light that splits up into beams of different wavelengths when it passes through a prism. This is part of our development as *Homo sapiens*. Since the dawn of mankind, we passed through many prisms and, on the other side, we proudly perceived ourselves wiser than before.

This is only a hallucination. What we gained was not wisdom or intelligence, but a new tool: a mace to kill a mammoth, a flint to slice it, a fire to bake it, writing to record a recipe. And as the artefacts congregated, the environment changed, and the human world was split into tribes and civilisations and nation states. That is how it was when the age of flint tools ended and the bronze sword arrived, followed by the iron plough. That’s when an invention from Mainz—letterpress printing—started mass media and when another invention from the East—gunpowder—enabled Europe to conquer large parts of the world. Caravels, loaded to the waterline with novelties from foreign civilisations, sailed across oceans to enrich the Old World. Old empires were overthrown or declined, and new ones emerged from the ashes; in Wittenberg the one church was split, medieval alchemy disintegrated and the nascent modern science blinked in its fragments.

What we gained was not wisdom or intelligence, but a new tool: a mace to kill a mammoth, a flint to slice it, a fire to bake it, writing to record a recipe.

Alchemy mixed all the knowledge of the time—astrology, medicine, metallurgy, the seeds of chemistry and physics—with superstition and myths: The universe was created from the four basic elements—earth, water, fire, and air—enclosed in a great order wherein everything was connected to each other. “Wisdom has built her house; she has carved out her seven pillars” (Proverbs 9:1). Thus, to the seven celestial bodies belonged the seven days of the week, the seven metals, and the seven organs: Sun ruled over gold and the heart; under the influence of Moon was silver and the brain; to Mercury belonged metal of the same name and the lungs; in dominion of Venus there were copper and kidneys; Mars was the lord of iron and the gallbladder; under the sceptre of Jupiter were tin and the liver; and Saturn had power over lead and the spleen.

Alchemists would have written in their grant proposals that their ultimate goal was to find the Philosopher’s Stone, to create gold by transmuting another metal. They failed and while Europe suffered from the black death, alchemy did not help. After Marco Polo brought news from Persia about the production of zinc, and metallurgists mined bismuth, antimony and platinum, it became obvious that there were more than seven metals. The dogmas of alchemy shattered; modern science rose in its wake.

Millions of years have passed since flintstones, hundreds of years from the raiders of the Philosopher’s Stone. We know much more about natural laws, the universe and the events taking place in the human body, all by virtue of the modern science. Many novel artefacts have congregated, and our environment is changing again. We enter the prism once more, and we consider it a natural thing that we will be smarter than before on the other side. Yet, it is still nothing but a hallucination. It is just another tool in our hands: a heat-seeking missile instead of a mace; a computer instead of an abacus.

## Harmful effects of beneficial activities

In his film *2001: A Space Odyssey*, Stanley Kubrick condensed the million-year human journey in one single cut: a bone, the first human tool, is thrown up in the air and transforms into a spacecraft. The film was released in 1968, the year before the Moon landing. By setting its plot into 2001, Kubrick anticipated numerous technologies to have been discovered at that time. Today, half a century later, many of his ideas have come true, such as artificial intelligence. However, we are far away from the optimistic vision depicted in the film showing, for example, people of different nationalities meeting on the Moon to solve global issues.

Yes, a few Earth inhabitants built the Rosetta spacecraft and send it into dark space in the wake of a whizzing rock called 67 P/Churyumov–Gerasimenko (Fornasier et al, 2016). On the other hand, there are more and more Earthlings who firmly believe that the Earth is flat. People are still dying in wars, famines and from diseases that we have been able to prevent or cure for a long time. Thousands of species are going extinct, habitats are lost, the climate is changing drastically, corruption is spreading in many countries, economies are collapsing, and human rights, democracy and freedom are under threat.

Today’s scientists may only dream of meeting colleagues on the Moon. Instead, quite a few of them see human civilisation as a failed experiment that is coming to its inevitable end. But I believe that even the biggest sceptics among them use to read bedtime stories to their children about how good defeats evil in the end. For the sake of these children, scientists need to find a way out of the current global crisis. However, successful crisis management requires pre-evaluation of the crisis management team first.

## Ideal versus real science

So, what actually do scientists? They look for unexplained phenomena and try to find an explanation by way of designing and conducting experiments. Based on the results, they refute or confirm, or better said, do not refute their hypotheses to explain said phenomena. This is how the philosopher Karl Popper described ideal science. The reality is a bit different: I do not know how it is elsewhere, but a real scientist in Slovakia must do market research and fill out orders. A real scientist must write grant proposals with specific milestones and deliverables to be achieved. A real scientist needs to publish and be cited as much as possible. This forces the real scientist to focus on topics that easily lead to publications rather than studying phenomena that pique his or her interest. The pressure to publish original results means that most publications are not verified or refuted, and increases the risk that some take a shortcut and falsify their results. According to a 2016 study, more than 70% of published scientific results cannot be reproduced (Baker, 2016).

For alchemists, it was not easy to publish their works: back then, printing books was a luxury that only the wealthy could afford. Moreover, there was a risk that the books would end up in the fire of the Inquisition. When books finally made it onto the shelves of university libraries, they were a treasure for students to look at with awe. Today, millions of scientific articles are available on the Internet—the Pubmed database of the US National Center for Biotechnology Information (NCBI) adds several articles every minute just in the fields of biology and medicine alone. “Several lives would not be enough”, the poet Francis Ponge observed almost a century ago, and continued: “Amidst the enormous and increasing extent and quantity of knowledge acquired by each science, we are lost. The best course to take is therefore to consider all things as unknown, and to walk or lie down in the woods or on grass, and start again from the beginning” (Introduction to the Pebble).

Those countless articles sometimes have so many authors that even they themselves do not read it all but only with the parts they contributed. Scientific works are all the more incomprehensible for the lay public, and many journals are still behind a paywall. On the other hand, disinformation portals are open for anyone and the ‘opinions’ they spout are easy to grasp and understand. The fact that more and more people today believe nonsense is also a consequence of this setup: the reliable information is hard to find and comprehend; the harmful one is free and simple.

The fact that more and more people today believe nonsense is also a consequence of this setup: the reliable information is hard to find and comprehend; the harmful one is free and simple.

Increasing need for grants, publications and citations make contemporary scientists stressed, anxious and performance-oriented (Gin et al, 2021). These hindrances do not help to understand the diversity and complexity of the world. However, those are just minor shortcomings, reviewer #1 of the crisis management plan would remark, and to change it should not be an insurmountable problem. Indeed, many scientists and science policymakers are trying to come up with solutions. For example, more and more scientific agencies no longer look at the h-indexes of applicants or the impact factors of journals but instead focus on the research per se. More funding agencies and philanthropies embrace and support true “blue skies” research to explore new uncharted territories or specifically focus their money on finding solutions to pressing humanitarian or environmental problems.

In contrast to those removable barriers, the very method whereby modern science had long time ago approached its mission may be seen as dogmatic in a medieval sense. And this is a problem requiring a major revision, as reviewer #2 might point out since it concerns a fundamental question that all scientists should ask themselves: how to benefit humanity? As a result, modern science, notwithstanding its impressive progress, has also brought negative by-effects. Now, scientists must find a solution to address the harmful consequences of their work.

## Repetitive wake-up calls

To cope with a crisis not only requires a crisis management team, but also, and above all, to analyse the problem itself. In the 17th century, René Descartes, a founder of modern philosophy and sciences, has posed man as a master of creation (“*maitre et proprietaire de la nature*”)—the one and only capable of reasoning—in contrast to animals, which Descartes denied a soul and which were in his view just living machines unable to think and feel (“*machina animata*”). But as Milan Kundera wrote in his novel *The Unbearable Lightness of Being*: “True human goodness, in all its purity and freedom, can come to the fore only when its recipient has no power. Mankind’s true moral test, its fundamental test (which lies deeply buried from view), consists of its attitude towards those who are at its mercy: animals. And in this respect mankind has suffered a fundamental debacle, a debacle so fundamental that all others stem from it” (translated from the Czech by Michael Henry Heim). Extinction of species and destruction of their habitats are direct consequences of Descartes’ primary conception. A way out for Kundera’s characters was to “step down from the road along which mankind—the master and proprietor of nature—marches onward”.

“Mankind’s true moral test, its fundamental test (which lies deeply buried from view), consists of its attitude towards those who are at its mercy: animals.”

The wrong course that modern science has taken could have been noticed from many warning signs even from within science itself. The last wake-up call is the New York Declaration on Animal Consciousness signed by many scientists suggesting the existence of consciousness not only in mammals and other vertebrates, but also in insects and molluscs, which is in a direct contradiction to mechanistically conceived organisms as Descartes defined it. But it was much earlier before the declaration, in the 19th century, when Charles Darwin revealed the scientific fundament for the human kinship with all forms of life on Earth. In his book *On the Origin of Species*, he argued that mankind is nothing but an ordinary component in the chain of creation, a mere subject to the same laws of evolution as all other organisms. By the way, Darwin and other epochal discoverers, such as Mendel or Faraday, were amateur or self-made scientists who never held a scientific grant.

In the 20th century, the physician Albert Schweitzer postulated his theory of reverence for life (“Ehrfurcht vor dem Leben”) with which he, in my opinion, touched the eternally sought-after universal principle—an equation of everything. Its meaning was crystal clear: “I am life which wills to live, and I exist in the midst of life which wills to live”.

Every period has had its scientists who understand that man would not be able to survive without his fellow inhabitants, but they have always been drowned out by those who see man as the master of creation. There are always more important concerns, such as the gross national product, new roads, more housing, and meals, than protecting the environment. Such an attitude stems from a lack of comprehension of how closely and inextricable mankind is tied to nature. This prolonged spasm of ignorance only strengthens baseless human feelings of their uniqueness. Our private habits, social practices and global policies thus paralyse all attempts to implement international environmental protection and recovery programmes.

Our private habits, social practices and global policies thus paralyse all attempts to implement international environmental protection and recovery programmes.

## Step down without losing science

Modern science has two options. First, scientists may systematically record all the global changes associated with the adverse impacts of human activities on our ecosystem: precisely measure the extend of global warming; carefully observe the decay of disappearing habitats; evaluate in detail all species threatened by extinction; and produce new scientific terms to describe these phenomena, such as the “Anthropocene” (Laurance, 2019). In a nutshell, modern science may chronicle the end of our current civilisation. In this way, it will become postscience similar to postmodern introduced by the philosopher Jean-François Lyotard as the metaphor of the end of “grand narratives” (Holtz, 2020). Alternatively, scientists may correct the wrong course taken over centuries. This however requires two tasks: to propose solutions and to communicate them to the public and governments.

In due fact, many environmental scientists and their colleagues from other scientific areas have been proposing solutions for decades. For example, they insist on less logging and more planting; they propose to cultivate forests and use wood, which has a lighter environmental footprint, as a building material (Mishra et al, 2022). Environmental scientists call for less fishing (Sumaila et al, 2021); they ask for modifying our western style eating habits (Stull and Weir, 2023) and humbling our economic profit expectations (Otero et al, 2020); they work on recovering ecosystems and protecting endangered species and their environment (Loreau et al, 2022); they develop eco-friendly building materials (Peng et al, 2022) and sustainable energy technologies (Acevedo-Rueda et al, 2023); or they release seals from entanglements. All their efforts are aimed at achieving the major objectives of the crisis management plan: to slow down and potentially stop the environmental damages; to mitigate the consequences which we already face; and to teach how to cope with them.

Still, the second mission, that is, communication, is much harder. A change in human thinking and behaviours that have been imprinted throughout centuries cannot be expected to occur from one day to another. However, modern science must enter this narrow gate and call for “true human goodness”. Science can even argue through faith, namely via the New Testament parable of tenants who must pay rent to the owner of a vineyard (Luke 20:9), which is in striking contrast to the Old Testament perception of mankind ruling “over all the earth” (Genesis 1:26). And above all, science must stand on pure logic and reason.

## An island as an example for a planet

The second law of thermodynamics—formulated in the 19th century by Lord Kelvin and Rudolf Clausius— was discovered as a side effect of research on improving the efficiency of steam engines. As each machine transforms only a portion of energy into work—the rest is released as heat ‘waste’—a good engine design aims to waste as little energy as possible. If we regard life on Earth as such a machine, a reasonable engineer would strive for the highest possible efficiency. Being at the same time a component of the machine, this endeavour would confront us with the need to produce significantly less waste and recycle unavoidable garbage. In other words, science must teach people that constant economic growth is not a fundamental given. An experiment on the Swedish island of Gotland may serve as a model for solving problems we face on a global scale (Wald, 2022). In brief, a team of researchers, together with a local company, launched a recovery project to recycle the islanders’ urine in the form of fertilisers. The result should be less use of synthetic fertilisers and less human urine released into the seas.

Half a millennium ago, one of the old worlds transformed with the insights of Nicolaus Copernicus and Galileo Galilei—who confirmed Copernicus’ earlier observation that the Earth revolved around the sun—and the human view of the universe changed from geocentric to heliocentric. Today, the anthropocentric universe must be replaced by a zoicentric universe (zoi, Greek ζωή – life). Mankind must “step down from the road along which” all living beings walk. Step down without losing science because science can determine what will be the face of a new world awaiting on the other side of the prism. Thus, the final decision on the crisis management team should be definitively positive about science.

Today, the anthropocentric universe must be replaced by a zoicentric universe (zoi, Greek ζωή – life).

Nevertheless, are contemporary scientists able to fulfil this demanding mission? After all, we have to write orders, reports, project proposals, publications, and other duties. Until one day we no longer remember the colour of our child’s eyes. That’s the moment to honestly ask ourselves as in the song from Rush *The Stars Look Down*: “What is the meaning of this? What are you trying to do? And the stars look down”.

### Supplementary information

Peer Review File
