# Supplementary material for: And the stars look down: science beyond the finite: From prehistorical science to postscience
Source: EMBO Rep. 2024 Jul 5;25(8):3177–81. doi: 10.1038/s44319-024-00202-w (PMC11315908; doi:10.1038/s44319-024-00202-w)
Supplement: Supplementary file 1 — Peer Review File [file 44319_2024_202_MOESM1_ESM.pdf]

EMBO reports

And the stars look down: Science beyond the finite

Vladimir Leksa

Corresponding author(s): Vladimir Leksa (vladimir.leksa@savba.sk)

Review Timeline:

Submission Date:	12th May 24
Editorial Decision:	16th May 24
Revision Received:	22nd May 24
Accepted:	28th Jun 24

Editor: Holger Breithaupt

Transaction Report: This article was editorially reviewed.
